# Racial, Ethnic, and Nativity Differences in Mental Health Visits to Primary Care and Specialty Mental Health Providers: Analysis of the Medical Expenditures Panel Survey, 2010–2015

**DOI:** 10.3390/healthcare6020029

**Published:** 2018-03-22

**Authors:** Audrey L. Jones, Susan D. Cochran, Arleen Leibowitz, Kenneth B. Wells, Gerald Kominski, Vickie M. Mays

**Affiliations:** 1 Informatics, Decision-Enhancement and Analytic Sciences Center (IDEAS 2.0), VA Salt Lake City Health Care System, Salt Lake City, UT 84148, USA; 2 Department of Internal Medicine, University of Utah School of Medicine, Salt Lake City, UT 84132, USA; 3 Department of Epidemiology, Jonathan and Karin Fielding School of Public Health, University of California, Los Angeles (UCLA), CA 90095, USA; cochran@ucla.edu; 4 Department of Statistics, University of California, Los Angeles, CA 90095, USA; 5 UCLA Center for Bridging Research Innovation, Training and Education for Minority Health Disparities Solutions, Los Angeles, CA 90095, USA; mays@ucla.edu; 6 UCLA Luskin School of Public Affairs, Los Angeles, CA 90095, USA; arleen@ucla.edu; 7 UCLA David Geffen School of Medicine, Los Angeles, CA 90095, USA; kwells@mednet.ucla.edu; 8 UCLA Center for Health Services and Society, Semel Institute for Neuroscience and Human Behavior, Los Angeles, CA 90095, USA; 9 Department of Health Policy and Management, Jonathan and Karin Fielding School of Public Health, University of California, Los Angeles, CA 90095, USA; kominski@ucla.edu; 10 UCLA Center for Health Policy Research, Los Angeles, CA 90024, USA; 11 Department of Psychology, University of California, Los Angeles, CA 90095, USA

**Keywords:** healthcare disparities, mental health services, primary health care, African Americans, Hispanic Americans

## Abstract

Background. Black and Latino minorities have traditionally had poorer access to primary care than non-Latino Whites, but these patterns could change with the Affordable Care Act (ACA). To guide post-ACA efforts to address mental health service disparities, we used a nationally representative sample to characterize baseline race-, ethnicity-, and nativity-associated differences in mental health services in the context of primary care. Methods. Data were obtained from the Medical Expenditures Panel Survey (MEPS), a two-year panel study of healthcare use, satisfaction with care, and costs of services in the United States (US). We pooled data from six waves (14–19) of participants with serious psychological distress to examine racial, ethnic, and nativity disparities in medical and mental health visits to primary care (PC) and specialty mental health (SMH) providers around the time of ACA reforms, 2010–2015. Results. Of the 2747 respondents with serious psychological distress, 1316 were non-Latino White, 632 non-Latino Black, 532 identified as Latino with Mexican, Central American, or South American (MCS) origins, and 267 as Latino with Caribbean island origins; 525 were foreign/island born. All racial/ethnic groups were less likely than non-Latino Whites to have any PC visit. Of those who used PC, non-Latino Blacks were less likely than Whites to have a PC mental health visit, while foreign born MCS Latinos were less likely to visit an SMH provider. Conditional on any mental health visit, Latinos from the Caribbean were more likely than non-Latino Whites to visit SMH providers versus PC providers only, while non-Latino Blacks and US born MCS Latinos received fewer PC mental health visits than non-Latino Whites. Conclusion. Racial-, ethnic-, and nativity-associated disparities persist in PC provided mental health services.

## 1. Background

Mental health services are purported to be available in United States (US) primary care settings; however, mental disorders still often go untreated, especially among racial and ethnic minority populations. Primary care is considered the de facto provider of mental health services as most patients receive at least some of their mental health care from primary care (PC) providers [1,2]. Indeed, PC providers are often more accessible to patients than mental health specialists and visiting a PC provider for mental health may carry less stigma than specialty mental health (SMH) settings. PC providers are responsible for screening their patients for mental health disorders, providing medication management, and referring patients to mental health specialists for treatment of more serious disorders [3]. These PC responsibilities have grown over time with the availability and response to medication treatments, development of brief therapies for delivery in PC settings, and dissemination of guidelines to direct provider behaviors [4,5]. Despite these advances, only half of all patients with mental health disorders receive any mental health services [6]. Moreover, there are widespread racial, ethnic, and nativity disparities regarding the receipt of mental health services in the US [7,8,9]. 

The Institute of Medicine’s 2003 report, “Unequal Treatment: Confronting Racial and Ethnic Disparities in Health Care”, provides a useful framework for understanding disparities in mental health services [10]. The Institute of Medicine committee found that healthcare disparities are shaped by the organization, financing, and regulatory environment that affects patients’ access to care, factors within the clinical encounter that contribute to poor quality of care for vulnerable versus non-vulnerable populations (e.g., provider discrimination or bias, patient-provider mistrust, and clinical uncertainty). In the case of mental health, several studies have documented racial, ethnic, and nativity differences in receipt of mental health services and the consequences of poorly managed mental health disorders within minority populations. For instance, Blacks and Latinos are less likely than Whites to receive any mental health treatment [11,12,13,14], especially evidence-based treatment [15,16,17,18]. When US nativity is assessed, foreign born Black and Latino immigrants are even less likely than US born Blacks and Latinos to receive needed mental health services [7,8,19]. These gaps in services delivery contribute to detrimental outcomes, such as disruptions in education and employment; reductions in quality of life; greater severity and persistence of mental disorders; and increased risks for chronic medical conditions, premature mortality, and suicide [20,21,22,23,24,25,26,27,28,29].

Because more patients receive mental health services from PC providers than SMH providers, primary care is an optimal setting to begin addressing racial, ethnic, and nativity disparities. Although patients may prefer the PC setting, Black and Latino groups experience poorer access to PC providers than Whites as a result of insurance and cost barriers, lack of a usual PC provider, and PC provider shortages in neighborhoods with large concentrations of racial/ethnic minorities [6,30,31,32,33]. Even when Blacks and Latinos use PC, their mental health disorders may remain untreated or poorly managed in this setting [34,35,36,37,38,39]. This occurs when primary care providers fail to diagnose mental health disorders, are not trained to deliver counseling-based therapies favored by racial/ethnic minorities, or lack access to mental health specialists for consultation and referral [37,40,41,42,43,44].

The majority of prior studies on racial, ethnic, and nativity disparities in mental health services in PC settings are based on data collected before 2010, prior to the passing of the Patient Protection and Affordable Care (ACA). With health insurance expansions and mental health coverage requirements rolling out in 2014, the ACA was expected to provide new access to PC providers and reduce cost barriers to mental health treatment for Black and Latino minorities [45,46]. In addition, the ACA called for a redesign of PC with a focus on the integration of physical and mental health services under patient-centered medical homes and accountable care organization reforms [46,47,48]. The few published investigations into mental health services use around the time of the ACA have not specifically examined PC-provided mental health services [9,49,50,51]. Moreover, limited research in this area has not accounted for heterogeneity within the Latino population with respect to both US versus foreign/island nativity and country of origin which, based on analyses of Black populations in the National Survey of American Life, can differ by nativity [8]. Understanding barriers to PC and shortcomings within this setting for specific populations could help to design interventions aimed at reducing mental health service disparities in the post-ACA era as well as provide evidence to policymakers as they shape healthcare reforms.

## 2. Aims

This study used nationally representative data from the Medical Expenditures Panel Survey to characterize baseline patterns of mental health services use in the context of primary care for groups differing in race, ethnicity, and US versus foreign/island nativity at the start of ACA reforms (2010–2015). Specifically, we estimated disparities in having any PC visit and, among patients utilizing PC, the receipt of mental health services from PC or SMH providers. We also examined racial, ethnic, and nativity differences in the types of providers seen (PC only, SMH only, PC and SMH) and numbers of mental health visits to PC and SMH providers. Recognizing diversity within the growing Latino population, we estimated disparities separately for Latinos of Mexican, Central American, and South American heritage versus Puerto Rican, Cuban, and other Caribbean island origins, as well as US versus foreign/island born nativity.

## 3. Methods

### 3.1. Design and Setting

Data were obtained from the Household Component of the Medical Expenditures Panel Survey (MEPS), a two-year panel study of healthcare use, satisfaction with care, and costs of services in the US [52]. We pooled data from six longitudinal waves (14–19) of participants to increase precision of the disparity estimates and explore potential interactions of race/ethnicity with nativity.

### 3.2. Analytic Sample

The full MEPS sample was restricted to non-Latino Black, non-Latino White, and Latino adults who participated in the full two years of MEPS between 2009 and 2015 and who met criteria for serious psychological distress on the K-6 measure of psychological distress [53,54]. The K-6 consists of six items that measure recent symptoms of anxiety and depression over the past month: “During the past 30 days, how often did you feel… nervous, hopeless, restless or fidgety, so sad that nothing could cheer you up, everything was an effort, worthless?” Participants chose from Likert response categories ranging from “all of the time” (4) to “none of the time” (0), producing a total score ranging from 0–24. We used a standard cutoff of 13 or higher to identify adults with serious psychological distress at the round two interview [55,56]. After excluding data from 3% of participants with missing data on key study variables, the analytic sample included 2747 adults aged 18 or older with serious psychological distress.

### 3.3. Measures

#### 3.3.1. Race, Ethnicity, and Nativity

We used responses to questions on race, ethnicity, and country of origin to group participants based on their shared racial/ethnic heritage. Non-Latino adults were grouped according to their self-reported race: White or Black. Those who reported Hispanic or Latino ethnicity were divided into two groups according to shared regional heritage: Mexico, Central America, or South America (“MCS”) or the Spanish-speaking Caribbean (Puerto Rico, Cuba, Dominican Republic, Other Non-MCS Latino heritage, “Caribbean”). Adults born outside the US mainland were coded as foreign/island born.

#### 3.3.2. Medical and Mental Health Services

Participants reported on healthcare visits from 2010 to 2015. The household informant described all household members’ outpatient and office-based visits, including the members’ reason for the visit, type of healthcare provider seen, and the type of services received during the visit. We aggregated responses from the round 3 to round 5 interviews (covering approximately 15 months) to determine mental health visits in the year following the round 2 assessment of psychological distress (“Year 2”).

A “PC visit” was defined as an outpatient or office-based visit to a nurse practitioner, physician’s assistant, or physician specializing in general practice, family medicine, or internal medicine. A “mental health visit” included outpatient or office-based visits where the reported reason for the visit included a mental health or substance use condition (Clinical Classification Code: 650–652, 654–662, 670) [57] or where the visit included counseling/psychotherapy services or alcohol/drug treatment.

We examined the types of providers that patients saw for mental health. A PC-provided mental health visit was defined as a mental health visit to a PC provider, while SMH visits included those to a psychiatrist, psychologist, or social work provider. “Other” visits included mental health visits to a provider that did not meet the study definition of PC or SMH. This could include medical specialists (e.g., cardiology, obstetrics-gynecology) and complementary alternative medicine providers. We created a categorical variable to indicate the type(s) of providers seen for mental health during the study period: PC provider only, SMH provider only, both PC and SMH providers, or other types of providers (non-PCP, non-SMH).

Because some PC patients received psychiatric medications but did not report a mental health visit, we separately examined medication treatments for mental health conditions. Medications were coded as “mental health” if the prescription belonged to a psychotropic drug class and respondents reported the reason for the prescription was to treat a mental health or substance use condition.

#### 3.3.3. Covariates

We estimated racial, ethnic, and nativity differences in mental health visits after controlling for measured indicators of clinical appropriateness, need for services, and preferences for care. These included: age, gender, marital status (single and never married, married or cohabitating, previously married), ratings of mental health status (i.e., “How would you rate your mental health today?”), number of self-reported chronic medical conditions, physical health limitations, the SF-12 physical health composite score, and self-reliant healthcare attitudes (see Table 1). We controlled for survey year (2014–2015 versus 2010–2013) to account for potential changes in PC and SMH visits following the ACA insurance expansions. We also examined healthcare access variables, including types of insurance, education, household income relative to poverty level, geographic region of the US, urban versus rural residence, and usual source of care.

### 3.4. Statistical Analysis

All statistical analyses were conducted in Stata 13.0 [58]. We applied survey weights to account for participants’ differential selection into MEPS, dropout during the two-year follow-up period, and post-stratification to provide estimates generalized to the US population. Survey weights included in the MEPS public use files were adjusted to account for the pooling of participants from panels 14–19 and standard errors were estimated using a Taylor series approximation, which accounts for the complex sampling design. *p*-Values less than 0.05 were considered statistically significant throughout.

Cross-tabulations were used to assess bivariate associations of race/ethnicity with sociodemographic characteristics, medical and mental health visits to PC and SMH providers, and mental health prescriptions. We also estimated the percent of Black, White, MCS, and Caribbean patients who reported a mental health visit to PC providers only, SMH providers only, PC and SMH providers, other types of providers as well as the mean number of mental health visits to MH and SMH providers. The significance of racial/ethnic differences in sociodemographic characteristics and mental health visits were tested using Wald chi-square and analysis of variance tests.

Multivariable logistic regression methods were used to estimate racial/ethnic disparities in the probability of a PC visit as well as probabilities of mental health services use conditional on any PC visit. The multivariable model controlled for mental health need and healthcare attitude covariates. This approach is similar to approaches employed by other researchers applying the definition of a healthcare disparity from the Institute of Medicine’s “Unequal Treatment” report in studies of mental health services use [12,16,59]. Multivariable multinomial regression methods were used to examine racial/ethnic differences in the types of mental health providers seen (PC only, SMH only, PC and SMH, or other). Then, negative binomial regression methods were used to estimate racial/ethnic differences in frequency of mental health visits to PC and SMH providers. The race/ethnicity coefficients from the negative binomial models were converted to predictive margins to aid interpretation [60].

In sensitivity analyses, we explored potential interactions of US versus foreign/island nativity with race/ethnicity and tested whether the addition of healthcare access covariates explained racial/ethnic disparities in medical and mental health visits to PC and SMH providers.

## 4. Results

### 4.1. Characteristics of Non-Latino White, Non-Latino Black, and Latino Adults with Serious Psychological Distress

The sample included 2747 adults with serious psychological distress, including 1316 non-Latino Whites, 632 non-Latino Blacks, 532 MCS Latinos, and 267 Caribbean Latinos; 525 were foreign/island born. There were significant racial/ethnic differences across most indicators of mental health need (Table 1). Black and MCS adults were younger than Whites on average and less likely to rate their mental health as “fair” or “poor”. Half of the adults in the MCS and Caribbean Latino groups were foreign/island born compared to less than 5% of Whites and Blacks. In addition, there were ethnic differences across indicators of physical health status (e.g., number of chronic health conditions, physical health limitations) for MCS Latinos versus Whites, suggesting that some MCS Latinos experienced better physical health than Whites.

There were also significant racial/ethnic differences across indicators of healthcare access. Compared to White participants, a greater percentage of Black and MCS participants reported a high school or lower education level and lived in households near the Federal Poverty Level. MCS Latinos were more likely than all other racial/ethnic groups to be uninsured and less likely to have a usual source of care. In contrast, Caribbean Latinos and non-Latino Blacks were more likely than Whites to be covered by Medicaid than private insurance. Additionally, each of the racial/ethnic minority groups were geographically clustered, with over half of non-Latino Blacks and MCS Latinos living in the Southern and Western regions of the US, respectively, and with Caribbean Latinos primarily residing in the Northeast and Southern US regions.

### 4.2. Primary Care and Mental Health Service Visits among Non-Latino White, Non-Latino Black, and Latino Adults with Serious Psychological Distress

Each of the racial/ethnic minority groups were less likely than Whites to visit a PC provider (75–80% vs. 85%; Table 2). Of those who utilized primary care, Black, MCS Latino, and Caribbean Latino groups appeared less likely than Whites to receive a PC mental health visit (18%, 19%, 19% vs. 24%). MCS Latinos were less likely to visit SMH providers (20% vs. 28%), while all Black and Latino groups were less likely to receive a mental health prescription (44%, 39%, 54% vs. 63%). In unadjusted analyses, there were no racial/ethnic differences in the types of providers seen for mental health and no differences in the numbers of MH visits to PC and SMH providers.

### 4.3. Adjusted Racial, Ethnic, and Nativity Disparities in the Likelihood of Primary Care and Specialty Mental Health Visits

After controlling for measured indicators of healthcare needs and attitudes, non-Latino Blacks, MCS Latinos, and Caribbean Latinos were less likely than non-Latino Whites to receive any PC visit (Table 3: adj. ORs = 0.54, 0.57 and 0.52, respectively). Participants who were older, female, and those with chronic conditions were more likely than others to use PC. There were no differences in PC visits based on foreign/island nativity or MEPS year.

Of those with any PC visit, some racial/ethnic disparities in mental health services remained. Black minorities were less likely than Whites to receive a PC mental health visit (adj. OR = 0.65) and less likely to receive a mental health prescription (adj. OR = 0.40). MCS Latinos and foreign/island born adults were less likely than White and US born adults to receive a mental health prescription (adj. ORs = 0.57 and 0.56, respectively). Self-rated mental health and number of chronic medical conditions were associated with increased likelihoods of all types of mental health services. In addition, mental health prescriptions were more common in 2014–2015 compared to 2010–2013 (adj. OR = 1.35).

In sensitivity analyses that considered interactions between race/ethnicity and nativity, US born Black and Caribbean Latino groups were less likely than US born Whites to receive a PC mental health visit (adj. ORs = 0.67 and 0.44, respectively). In contrast, foreign born MCS Latinos were less likely than US born Whites to receive an SMH visit (adj. OR = 0.44; not shown in table). These patterns of racial, ethnic, and nativity disparities were unchanged after controlling for measured indicators of healthcare access (not shown in table).

### 4.4. Adjusted Racial, Ethnic, and Nativity Differences in Types of Mental Health Visits

Among those with any mental health visit, Caribbean Latinos were more likely than Whites to visit SMH providers only versus PC providers only (Table 4; Adjusted Relative Risk Ratio [adj. RRR] = 3.43). Caribbean Latinos were also more likely than Whites to receive care from SMH and PC providers versus PC providers only (adj. RRR = 3.16) and less likely to receive care from other medical providers versus PC providers only (adj. RRR = 0.24). Foreign/island born adults were more likely than US born adults to receive mental health services from other medical providers versus PC providers only (adj. RRR = 5.36). None of the Black-White and MCS Latino-White comparisons in types of mental health providers were statistically significant.

### 4.5. Adjusted Racial, Ethnic, and Nativity Disparities in Numbers of Mental Health Visits

In the adjusted models, Black minorities received fewer PC mental health visits than Whites (Table 5; adj. difference = −1.60 visits). There were no overall Latino-White or nativity-associated differences in numbers of mental health visits to PC or SMH providers. However, when interactions of foreign/island nativity with race/ethnicity were included in the models, US born MCS Latinos reported fewer PC mental health visits than US born Whites (adj. difference = −1.90 visits; not shown in table).

## 5. Discussion

This study provided baseline estimates of racial, ethnic, and nativity disparities in mental health visits to PC and SMH providers during the initial rollout of ACA reforms. We found that Black and Latino minorities were less likely than Whites to receive any PC visit. But even when these groups did use primary care, US born Blacks and Caribbean Latinos were less likely than Whites to receive a PC mental health visit; MCS Latino immigrants were less likely than US born Whites to visit SMH providers; and non-Latino Black, MCS Latino, and foreign/island born minorities were less likely than White and US born groups to receive mental health prescriptions. In contrast to prior studies which found that Black and Latino minorities were more likely than Whites to rely on primary care for their mental health treatment [61,62,63], none of the treated racial/ethnic minority groups were more likely than Whites to visit PC providers only. Blacks and US born MCS Latino received fewer numbers of PC mental health visits than non-Latino Whites. Collectively, the study’s findings illustrate persistent gaps in PC-provided mental health services around the time of ACA reforms. 

Since 2014, the ACA has reduced the numbers of Black and Latino adults without insurance and this was hoped to significantly impact the utilization of mental health services for racial/ethnic minorities [50,64]. While the current study did not longitudinally assess changes that might have occurred with insurance expansions, the lack of PC-provided mental health services within Black and Latino populations in the face of increased access to primary care services raises concerns. Our findings are somewhat aligned with a prior study of health insurance coverage conducted by Breslau and his colleagues in which racial/ethnic minorities did not equally benefit from the ACA provision that allowed children to stay on a parent’s health insurance plan through age 26 [65]. Findings from the current study suggest the need for vigilance as to whether important opportunities to assess, diagnose, and coordinate care for Black and Caribbean Latino patients are being adequately addressed or missed in the PC setting [66]. While more than 80% of Blacks and Caribbean Latinos with mental health needs reported any medical care visit, fewer than 20% of these Black and Caribbean Latino patients reported a PC mental health visit.

Several factors may contribute to racial, ethnic, and nativity disparities in PC-provided mental health services, including mistrust of PC providers or lack of time for providers to attend to mental health concerns because of the complexity of chronic care issues sometimes seen in these populations [37,67]. In addition, PC providers who serve racial/ethnic minority patients sometimes report clinical uncertainty in diagnosing and treating mental health disorders as well as difficulty in delivering high-quality care to their patients [41,68]. As Black and Latino minorities gain access to PC providers under the ACA, our findings, in combination with those from prior studies, underscore a need to improve the PC management of mental health disorders for these groups in particular [37]. Strategies to eliminate disparities in the PC setting may require efforts such as adequate time in appointments, training PC providers in culturally specific approaches of assessing and diagnosing mental disorders, or increasing diversity in patient-centered medical home teams to include patient navigators or religious leaders [69]. 

Among Latinos, there is clearly a cultural component that influences mental health services use, one which differs among Latino populations with diverse heritages and levels of acculturation. Our finding that foreign born MCS Latinos (but not US born MCS Latinos) were less likely than US born Whites to visit SMH providers suggests there are unique barriers to SMH providers for MCS immigrants. Foreign born MCS Latinos may experience greater stigma concerns towards treatment of mental health disorders than US born MCS Latinos, contributing to their reluctance to use SMH-provided services [70]. Additionally, foreign born MCS Latinos may not view the medical provider as the primary resource for handling mental health problems [69,71]. As shown by Villatoro and colleagues, the more embedded Latinos were in their family networks, the greater the expectation for using religious leaders for mental health problems [71]. A third possibility for our findings is that foreign born MCS Latinos may live in racially/ethnically segregated neighborhoods where there are shortages of SMH providers [72], particularly ones who speak Spanish. Since our findings indicated no racial/ethnic differences in the number of SMH visits once initial care was established, efforts to address Latino-White disparities in SMH settings may need to attend to help-seeking behaviors in immigrant populations as well as supply side obstacles. 

Our findings, which suggest that only a small percentage of patients received mental health visits to PC and SMH providers, implies that challenges to physical and mental health services integration [41,44] persisted during the early years of ACA implementation despite greater access to health insurance benefits. These findings are noteworthy because prior studies have found that integrated approaches to treatment, such as the collaborative care model, are successful for treating mental health disorders in racial and ethnic minority populations [73,74,75]. State adoption of medical homes have the potential to improve the integration and coordination of services for patients with physical and mental disorders [76,77]. However, the majority of medical home demonstration projects have been conducted in PC settings which might miss patients with serious mental illness who receive all of their care in SMH settings [78]. Given that Caribbean Latinos are more likely than Whites to be treated by only SMH providers, there is a need to evaluate the effectiveness of integrated care approaches, such as medical homes, in healthcare settings where racial and ethnic minorities receive a majority of their mental health care [79]. 

Our findings should be considered in light of the study’s limitations. First, this study was unable to assess perceived need for treatment, which could explain our observation of low rates of mental health visits among racial/ethnic minority groups [80]. Related, our estimates of mental health visits among Blacks and Latinos may be conservative if participants did not report mental health reasons for their PC visit due to stigma or other concerns. Second, adults who were homeless, institutionalized, or living in military quarters were excluded from MEPS, limiting the generalizability of findings for those groups who often have higher rates of serious mental illness [81,82,83,84]. Third, we were unable to examine mental health visits based on Latino participants’ specific country of origin (e.g., Cuba, Puerto Rico, El Salvador). Finally, this study was not powered to longitudinally assess changes in racial, ethnic, or nativity disparities in PC provided mental health services that may have corresponded with the rollout of specific ACA provisions between 2010 and 2015. Despite limitations, this study’s estimates of Black-White, MCS Latino-White, and Caribbean Latino-White disparities serve as a baseline for comparison throughout the ACA and beyond. The importance of these findings is underscored by the detrimental consequences of untreated mental health disorders within Black and Latino populations, as well as evidence that racial/ethnic disparities in mental health service use may not be solved by the initial health reforms geared to expand insurance coverage [51].

## 6. Conclusions

Our study contributes new findings on racial, ethnic, and nativity differences in patterns of PC-and SMH-provided mental health services at the time of the rollout of the ACA legislation and subsequent to the Mental Health Parity Act. The ACA has reduced financial barriers to mental health treatment and has provided enhanced access to PC providers who provide the majority of mental health care. Our findings show that, despite the decrease in financial barriers, significant gaps in mental health services remain across racial, ethnic, and nativity groups. The extent to which primary care will serve as an effective source for mental health services for racial/ethnic minorities remains uncertain. As health reforms continue to evolve, attention to racial, ethnic, and cultural factors that promote or limit the delivery of mental health services for specific minority groups is needed to ensure that any proposed changes do not inadvertently contribute to a worsening of mental health disparities [65].

## Figures and Tables

**Table 1:** Sociodemographic Characteristics of Non-Latino White, Non-Latino Black, and Latino Adults with Serious Psychological Distress, 2010–2015.

Sociodemographic Characteristics	Non-Latino		Latino		Overall Tests of Differences	
	White *n* = 1316	Black *n* = 632	Mexico, Central or South America *n* = 532	Caribbean *n* = 267		
**Year 1 Mental Health Need Indicators**	**% (SE)**	**% (SE)**	**% (SE)**	**% (SE)**	**F**	***p*-Value**
Age					2.33	0.02
18–34	25.1 (1.5)	27.1 (2.5)	32.7 (2.6)	28.2 (4.1)		
35–49	28.5 (1.5)	32.4 (2.4)	31.3 (2.6)	28.5 (3.2)		
50–64	35.6 (1.6)	30.9 (2.3)	23.4 (2.5)	35.3 (4.1)		
65+	10.9 (1.0)	9.6 (1.6)	12.6 (2.0)	8.1 (2.1)		
Female	61.2 (1.6)	66.0 (2.3)	58.5 (2.6)	65.1 (3.8)	1.59	0.19
Foreign/island nativity	3.7 (0.7)	4.2 (1.1)	52.8 (3.8)	55.8 (4.3)	200.92	<0.001
Marital Status					15.42	<0.001
Married	39.6 (1.7)	19.5 (2.1)	42.3 (3.7)	30.6 (3.7)		
Previously married	23.8 (1.5)	47.4 (2.6)	32.4 (2.9)	37.2 (4.3)		
Single, never married	36.6 (1.6)	33.1 (2.1)	25.3 (2.7)	32.1 (4.3)		
“Fair”/“Poor” mental health rating	55.0 (1.7)	46.4 (2.6)	36.4 (2.8)	57.0 (4.2)	12.09	<0.001
Number of chronic health conditions					4.96	<0.001
Zero	22.6 (1.3)	20.1 (2.6)	36.1 (2.7)	16.4 (3.1)		
One	23.5 (1.4)	22.8 (2.4)	19.3 (2.4)	21.4 (3.3)		
Two or more	53.9 (1.7)	57.1 (2.9)	44.6 (2.9)	62.2 (4.0)		
Physical health limitation	47.8 (1.7)	49.1 (2.7)	29.9 (3.4)	48.8 (4.6)	8.85	<0.001
Physical health functioning (mean, SE)	40.0 (0.5)	38.9 (0.8)	40.0 (1.1)	39.7 (1.3)	0.48	0.70
Healthcare Attitude						
No need for health insurance	3.6 (0.7)	5.1 (1.0)	6.9 (1.7)	3.6 (1.8)	2.04	0.11
Insurance not worth the costs	28.2 (1.7)	22.0 (2.3)	22.2 (2.5)	29.0 (4.4)	2.40	0.07
Likely to take risks	22.6 (1.4)	23.1 (2.3)	23.0 (2.1)	23.0 (3.8)	0.02	0.99
Overcome illness without medical help	16.7 (1.3)	13.1 (1.9)	12.2 (2.1)	11.5 (3.3)	1.89	0.13
MEPS Cohort 2009–2012 ^1^	64.3 (2.0)	63.8 (2.9)	31.8 (2.8)	36.0 (4.3)	0.42	0.73
**Year 1 Socioeconomic Status Indicators**	**% (SE)**	**% (SE)**	**% (SE)**	**% (SE)**	**F**	***p*-value**
More than high school education	48.3 (1.7)	35.7 (2.7)	31.0 (2.7)	44.4 (4.3)	11.80	<0.001
Type(s) of insurance						
Uninsured	25.2 (1.7)	32.2 (2.6)	36.9 (2.3)	25.1 (4.0)	6.23	<0.001
Private	39.0 (1.6)	21.7 (2.0)	27.5 (2.7)	20.7 (3.6)	19.16	<.001
Medicaid	25.1 (1.6)	36.7 (2.4)	28.1 (2.8)	44.0 (4.0)	12.32	<.001
Medicare	26.6 (1.4)	23.2 (2.2)	18.1 (2.5)	24.4 (4.0)	2.81	0.04
Geographic region					21.39	<0.001
Northeast	14.4 (1.8)	10.3 (1.7)	3.4 (0.9)	43.1 (5.3)		
Midwest	26.3 (2.1)	22.1 (2.8)	2.2 (1.5)	6.2 (2.3)		
South	37.9 (1.9)	55.7 (3.4)	6.1 (3.8)	38.1 (5.1)		
West	21.1 (1.8)	11.9 (2.4)	57.6 (5.6)	12.6 (2.8)		
Have usual source of care	80.1 (1.5)	76.2 (2.3)	68.0 (3.2)	82.0 (3.4)	5.65	0.001
Income below 133% FPL ^2^	37.8 (1.6)	56.1 (2.7)	45.1 (3.5)	51.4 (4.1)	15.58	<0.001

Source: Medical Expenditures Panel Survey, Panels 14–19 (collected 2009–2015). Sample includes adults aged 18 or older with serious psychological distress as indicated by a score of 13 or greater on the K-6 Measure of Psychological Distress. ^1^ Medical Expenditures Panel Survey Cohort. The year corresponds to the first year of a two-year longitudinal panel. For example, the 2009 cohort were assessed for sociodemographic characteristics in 2009 and service utilization was examined in 2010. ^2^ FPL = Federal Poverty Level.

**Table 2:** Primary Care and Mental Health Service Visits among Non-Latino White, Non-Latino Black, and Latino Adults with Serious Psychological Distress, 2010–2015.

Outcome Variables	Non-Latino		Latino		F	*p*-Value
	White *n* = 1316	Black *n* = 632	Mexico, Central or South America *n* = 532	Caribbean *n* = 267		
Year 2 Services Use	% (SE)	% (SE)	% (SE)	% (SE)		
Any primary care (PC) visit	85.4. (1.3)	78.5 (2.3)	74.4 (2.6)	79.6 (3.5)	7.63	<0.001
Of those with any PC visit (*n* = 2218):						
Any MH visit to PC provider	24.2 (1.7)	18.1 (2.2)	19.1 (2.9)	19.3 (3.4)	2.32	0.08
Any MH visit to SMH provider	28.2 (1.6)	27.7 (2.3)	20.1 (3.0)	34.6 (4.2)	2.67	0.04
Any MH prescription	62.6 (1.8)	44.0 (2.6)	39.1 (3.0)	53.6 (4.0)	24.42	<0.001
Types of mental health visits ^1^					1.44	0.17
PC provider only	33.1 (2.4)	24.9 (4.0)	31.3 (6.2)	18.8 (5.0)		
MH specialist only	46.2 (2.7)	52.3 (4.1)	41.9 (5.5)	63.6 (5.7)		
PC and SMH providers	15.4 (2.0)	16.6 (2.9)	18.4 (5.2)	15.2 (3.8)		
Other providers	5.2 (1.1)	6.3 (1.8)	8.4 (3.5)	2.4 (1.2)		
Number of MH visits to PC providers (mean, SE) ^2^	4.8 (1.0)	2.8 (0.3)	3.0 (0.4)	3.5 (0.5)	2.19	0.09
Number of MH visits to SMH providers (mean, SE) ^3^	12.6 (1.0)	10.0 (1.1)	14.3 (4.1)	10.9 (1.4)	1.29	0.28

Source: Medical Expenditures Panel Survey, Panels 14–19 (collected 2010–2015). Sample includes adults aged 18 or older with serious psychological distress as indicated by a score of 13 or greater on the K-6 Measure of Psychological Distress. Services use was assessed over 15 months in three interview rounds following the K-6 assessment (called Year 2). Abbreviations: MH = mental health, PC = primary care, SMH = specialty mental health. ^1^ Analysis conducted with subgroup of PC patients with any mental health visit (*n* = 2218). ^2^ Analysis conducted with subgroup of PC patients with any PC mental health visit. ^3^ Analysis conducted with subgroup of PC patients with any SMH provider visit.

**Table 3:** Adjusted Racial/Ethnic Differences in Primary Care and Mental Health Service Visits, Medical Expenditures Panel Survey, 2010–2015.

Independent Variables	Any Primary Care Visit	Among Adults with Any Primary Care Visit (*n* = 2218)		
		MH Visit with PC Provider	MH Visit with SMH Provider	MH Prescription
	Adj. OR (SE)	Adj. OR (SE)	Adj. OR (SE)	Adj. OR (SE)
Race/Ethnicity				
Non-Latino White	1.0	1.0	1.0	1.0
Non-Latino Black	0.54 (0.10) **	0.65 (0.12) *	0.85 (0.13)	0.40 (0.06) ***
MCS Latino	0.57 (0.13) *	0.82 (0.19)	0.95 (0.23)	0.57 (0.10) **
Caribbean Latino	0.52 (0.13) *	0.72 (0.19)	1.34 (0.34)	0.74 (0.16)
Foreign/island nativity	1.02 (0.20)	0.93 (0.22)	0.72 (0.18)	0.56 (0.13) **
Age	1.01 (0.01) *	0.99 (0.01)	0.99 (0.01) *	1.00 (0.01)
Female gender	1.95 (0.33) ***	0.99 (0.15)	1.24 (0.21)	1.74 (0.25) ***
Married	1.29 (0.21)	0.84 (0.12) *	0.57 (0.09) **	0.87 (0.10)
Mental health rated “fair” or “poor”	1.19 (0.20)	1.56 (0.25) **	3.44 (0.57) ***	2.56 (0.34) ***
Chronic health conditions				
Zero	1.0	1.0	1.0	1.0
One	1.65 (0.30) **	0.91 (0.21)	1.37 (0.33)	1.60 (0.34) *
Two or more	4.96 (1.01) ***	1.03 (0.24) ***	1.78 (0.39) **	1.98 (0.39) **
Physical health functioning	0.99 (0.01)	1.00 (0.01)	1.01 (0.01)	1.00 (0.01)
Self-reliant healthcare attitudes	0.49 (0.18)	0.74 (0.30)	0.33 (0.13) **	0.59 (0.18)
Year 2014–2015 ^1^	1.01 (0.18)	1.09 (0.20)	1.32 (0.19)	1.35 (0.19) *

* *p* < 0.05, ** *p* < 0.01, *** *p* < 0.001. Abbreviations: MCS = Mexican, Central American, or South American heritage, Caribbean = Puerto Rican, Cuban, Dominican, or Other Spanish-Speaking Caribbean heritage, FPL = Federal Poverty Level, Adj. OR = Adjusted Odds Ratio, SE = Standard Error. Source: Medical Expenditures Panel Survey, Panels 14–19 (collected 2010–2015). Sample includes adults aged 18 or older with serious psychological distress, assessed with the K-6 Measure of Psychological Distress. ^1^ Second year of a two-year longitudinal panel. The year corresponds to the outcome period when primary care and mental health visits were examined.

**Table 4:** Racial/Ethnic Differences in Type of Mental Health Providers and Number of Visits, Conditional on any Mental Health Visit.

Independent Variables	SMH vs. PC Only	PC and SMH vs. PC Only	Other vs. PC Only
	Adj. RRR (SE)	Adj. RRR (SE)	Adj. RRR (SE)
Race/Ethnicity			
Non-Latino White	1.0	1.0	1.0
Non-Latino Black	1.38 (0.38)	1.18 (0.40)	1.76 (0.84)
MCS Latino	1.33 (0.48)	2.07 (0.95)	0.65 (0.43)
Caribbean Latino	3.43 (1.43) **	3.16 (1.43) *	0.24 (0.17) *
Foreign/island nativity	0.59 (0.23)	0.41 (0.18)	5.36 (3.01) **
Age	0.99 (0.01)	0.99 (0.01)	1.01 (0.02)
Female gender	1.32 (0.32)	1.37 (0.42)	0.87 (0.34)
Married	0.63 (0.14) *	0.49 (0.15) *	0.64 (0.27)
Mental health rated fair/poor	2.22 (0.52) **	2.92 (0.98) **	1.37 (0.52)
Chronic health conditions			
Zero	1.0	1.0	1.0
One	1.44 (0.49)	0.64 (0.28)	1.24 (0.68)
Two or more	1.44 (0.52)	1.20 (0.49)	0.55 (0.29)
Physical health functioning	1.02 (0.01)	1.02 (0.01)	1.00 (0.01)
Self-reliant healthcare attitudes	0.35 (0.21)	0.38 (0.29)	4.43 (2.99)
Year 2014–2015 ^1^	1.24 (0.31)	1.09 (0.35)	0.90 (0.37)

* *p* < 0.05, ** *p* < 0.01, Abbreviations: PC = Primary Care, SMH = Specialty Mental Health, MCS = Mexican, Central American, or South American heritage, Caribbean = Puerto Rican, Cuban, Dominican, or Other Spanish-Speaking Caribbean heritage, Adj. RRR = Adjusted Relative Risk Ratio, SE Standard Error. Source: Medical Expenditures Panel Survey, Panels 14–19 (collected 2010–2015). Sample includes adults aged 18 or older with serious psychological distress as indicated by a score of 13 or greater on the K-6 Measure of Psychological Distress. ^1^ Second year of a two-year longitudinal panel. The year corresponds to the outcome period when primary care and mental health visits were examined.

**Table 5:** Racial/Ethnic Differences in Number of Mental Health Visits by Type of Provider.

Race/Ethnicity	PC Providers		SMH Providers	
	Mean (SE) ^1^	Δ ^2^ (SE)	Mean (SE)	Δ (SE)
Non-Latino White	4.51 (0.66)		10.67 (0.96)	
Non-Latino Black	2.91 (0.43)	−1.60 (0.60) **	9.09 (1.08)	−1.58 (1.18)
MCS Latino	3.56 (0.80)	−0.96 (0.91)	15.07 (3.86)	4.39 (4.00)
Caribbean Latino	3.66 (0.95)	−0.85(1.14)	10.30 (1.64)	−0.37 (1.80)

** *p* < 0.01. Abbreviations: PC = Primary Care, SMH = Specialty Mental Health, MCS = Mexican, Central American, or South American heritage, Caribbean = Puerto Rican, Cuban, Dominican, or Other Spanish-Speaking Caribbean heritage. Source: Medical Expenditures Panel Survey, Panels 14–19 (collected from 2010–2015). Sample includes adults age 18 or older with serious psychological distress as indicated by a score of 13 or greater on the K-6 Measure of Psychological Distress. ^1^ Number of mental health visit over 15 months. Means were predicted from negative binomial regression models that controlled for age, gender, marital status, mental health ratings, chronic health conditions, physical limitations, self-reliant healthcare attitudes, and survey year. ^2^ Adjusted difference in the mean number of mental health visits for each racial/ethnic group compared to Non-Latino Whites.
